# Rechargeable Zn^2+/^Al^3+^ dual-ion electrochromic device with long life time utilizing dimethyl sulfoxide (DMSO)-nanocluster modified hydrogel electrolytes†

**DOI:** 10.1039/c9ra06785j

**Published:** 2019-10-09

**Authors:** Hopmann Eric, Haizeng Li, Elezzabi Adulhakem Y.

**Affiliations:** Ultrafast Optics and Nanophotonics Laboratory, Department of Electrical and Computer Engineering, University of Alberta Edmonton Alberta T6G 2V4 Canada hopmann@ualberta.ca haizeng@ualberta.ca

## Abstract

Despite recent advances in hydrogel electrolytes for flexible electrochemical energy storage, ion conductors still exhibit some major shortcomings including low ionic conductivity and short lifetimes. As such, for applications in electrochromic batteries, a transparent, highly conductive electrolyte based on a dimethyl-sulfoxide (DMSO) modified polyacrylamide (PAM) hydrogel is being developed and implemented in a dual-ion Zn^2+^/Al^3+^ electrochromic device consisting of a Zn anode and WO_3_ cathode. Gelation in a DMSO : H_2_O mixed solvent leads to highly increased electrolyte retention in the hydrogel and prolonged life time for ionic conduction. The hydrogel-based electrochromic device offers a specific charge capacity of 16.9 μAh cm^−2^ at a high current density of 200 μA cm^−2^ while retaining 100% coulombic efficiency over 200 charge–discharge cycles. While the DMSO-modified electrolyte shows ionic conductivities up to 27 mS cm^−1^ at room temperature, the formation of DMSO : H_2_O nanoclusters enables ionic conduction even at temperatures as low as −15 °C and retention of ionic conduction over more than 4 weeks. Furthermore, the electrochromic WO_3_ cathode gives the device a controllable absorption with up to 80% change in transparency. Based on low-cost, earth abundant materials like W (tungsten), Zn (zinc) and Al (aluminum) and a scalable fabrication process, the introduced hydrogel-based electrochromic device shows great potential for next-generation flexible and wearable energy storage systems.

## Abstract 2

A novel electrochromic energy storage device based on a long-lifetime DMSO-modified hydrogel electrolyte.

## Graphical abstract

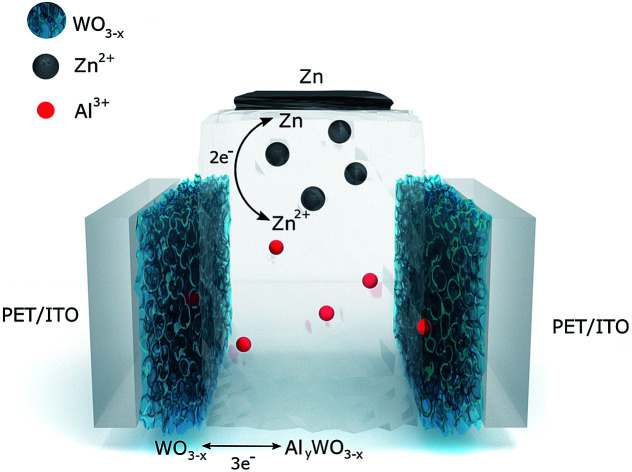

## Introduction

Taking advantage of the similarity of the physical and chemical mechanisms involved in electrochemically triggered chromatic response and capacitive ion storage has opened up a new frontier in multi-functional electrochromic energy-storing devices.^1–4^ Recently, it was shown that nano-engineered electrochromic materials allow independent control of visible and near-infrared (NIR) light.^5–7^ As promising as the field is, integration of light and heat management by electrochromic modulation and energy storage into the same device has been limited to electrochromic supercapacitors.^8–10^ In a new approach we have recently shown that battery functionality and the advantages of electrochromic electrodes can be combined in the same platform.^11^ For a safe and versatile battery architecture, improvements in electrode materials and electrolyte would be the natural progression. In the interest of eco-friendliness paired with novel functionality, new approaches to employ earth abundant and economically clean salts are needed.^11–14^

Zinc-ion batteries are the main contenders for lithium ion-based energy storage^15,16^ due to zinc's high charge capacity of 820 mA h g^−1^. The multivalent nature of zinc combined with its non-toxic nature have inspired a multitude of research in liquid and solid electrolyte-based devices.^15,17^ While lithium ion batteries pose as a potential fire and explosion hazard, zinc ion batteries are inherently inert and therefore safe to employ in personal electronic devices. For use in an energy storage device having multi-functional light and heat management properties, transparent electrodes are a requirement;^18^ therefore, inventive solutions are necessary to allow the use of opaque zinc electrodes in electrochromic batteries consisting of the traditional battery material zinc and electrochromic counter-electrodes based on transition metal oxides (TMO).

Within the class of TMOs, oxygen deficient tungsten oxide (WO_3_) has attracted most attention as an electrochromic pseudo-capacitor due to its high coloration efficiency and specific capacity.^19–21^ Previously, it was found that Zn^2+^ does not provide efficient intercalation into tungsten oxide.^11^ However, a dual-ion electrolyte can combine the redox reactions of zinc electrodes and the high coloration efficiency and of WO_3_ intercalated with trivalent aluminum ions.^22,23^ The comparably small ionic radius (53 pm) of Al^3+^ ions enables superior and reversible intercalation into WO_3_. Furthermore, the trivalent nature of Al^3+^ ions has been shown to allow faster intercalation than that of monovalent sodium ions and lithium ions,^24,25^ as well as exceptionally high charge capacities in WO_3_ (347 mA h g^−1^). Most importantly, the trivalent Al^3+^ intercalation into WO_3_ electrodes allows for increased lifetime of the electrochromic WO_3_ electrode due to the Al^3+^ ions shallow insertion depth and the multivalent electron transfer process during Al^3+^–WO_3_ intercalation.^26^ While these findings give insights into the mechanisms of intercalation itself, the applications are so far limited by the liquid nature of the electrolytes used.

Solid-state electrolytes (SSE), in various forms, have arisen mainly driven by safety concerns associated with liquid electrolyte in Li^+^ ion batteries.^27^ In comparison to their liquid counterparts, SSEs are said to inhibit dendrite growth, which could help increasing battery life.^28,29^ Moreover, SSEs permit wide ranges of operational temperatures, as well as an expanded span of working voltages and can be tailored towards their dielectric properties.^27,30,31^ The inherent leakage-free of the SSE allows for dense packing and ease of fabrication. While inorganic, ceramics-based ionic conductors have shown high promise, the inventive architecture of the electrochromic device requires an optically transparent electrolyte. However, ceramic electrolytes are mostly optically opaque, highly brittle, and may demand high pressure contact with the electrodes for ion transport. On the other hand, ionogels, which consist of ionic liquids having negligible vapor pressure at ambient conditions in polymer matrices, may possess high optical transparency and long lifetimes, but at the expense of low ionic conductivity and high cost.^32,33^ Nonetheless, for a high-performance electrochromic device, a highly conductive electrolyte is a basic requirement. Such properties are satisfied by a hydrogel as an ionic conductor.^3,34^

Hydrogels as ion conductors have emerged as key contenders in SSE zinc-ion batteries due to their high conductivity, mechanical flexibility, and low processing cost.^35–37^ Applications, such as batteries for smart wearables electronics, which require biocompatibility, are generally fulfilled by hydrogel electrolytes. However, a major limitation of PAM and other hydrogels (*e.g.* poly(ethylene-glycol), poly(ethylene-oxide)) is their short lifetime of ion conduction, which is rooted in the fast evaporation of water from the host matrix. A suggested solution was presented by exploiting the ionic hydration effect to bind water molecules to the host gel and thereby increasing the gel's water retention. Though, this mechanism has been mainly focused on lithium chloride (LiCl) as the hydrated salt, which limits the application to Li-ion based batteries energy.^38,39^ Hence, a long lifetime hydrogel with high electrolyte retention based on a different approach is needed for implementation in quasi SSE batteries.

Herein, we demonstrate a novel SSE dual-ion electrochromic device based on flexible zinc and WO_3_ electrodes and a PAM-hydrogel exhibiting remarkably long lifetime. To achieve a high electrochemical stability and to ensure increased electrolyte retention at ambient conditions, we utilized DMSO : H_2_O mixtures present in the gelation of the acrylamide. DMSO : H_2_O mixed solvents exhibit unique properties that are not found in the pure solvents, such as low freezing temperatures of down to 60 K and increased hygroscopicity.^40,41^ The significant changes in physical and chemical properties of the mixed solvent are based on the formation of nanoclusters triggered by the polar interaction of DMSO and water molecules. With the high level of electrolyte retention, the PAM hydrogel shows remarkable ionic conductivity of up to 27 mS cm^−1^, and weight retention of 72% after 28 days of storage under ambient conditions resulting in 5% retention of ionic conductivity. Moreover, the exceptional transparency (>95%) of the hydrogel on indium tin oxide (ITO) enables a dual-ion electrochromic energy storage device offering high areal capacities of up to 16.9 μA h cm^−2^ under 200 μA cm^−2^ discharge current with a capacity retention of over 60% after 200 cycles. More significantly, the exploitation of nanocluster formation allows for 20% energy storage capacity retention after 10 days under ambient conditions and enables device operation even at temperatures as low as −15 °C. The high flexibility of the gel electrolyte facilitates resilient devices withstanding bending and repeatedly applied pressure of 1.5 kPa.

## Experimental section

### Materials

Tungsten Powder, acrylamide monomer (AM), *N*,*N*′-methylenebisacrylamide (BAM), potassium persulfate (K_2_S_2_O_8_), zinc sulfate heptahydrate (ZnSO_4_·7H_2_O), aluminum trichloride (AlCl_3_) were purchased from Sigma-Aldrich at analytical grade and used without further purification. DMSO and hydrogen peroxide (30%, H_2_O_2_) were purchased from Fisher Scientific. ITO on glass (15 Ω sq^−1^) and ITO on PET (60 Ω sq^−1^) substrates were purchased from Sigma-Aldrich and cleaned with dish soap and de-ionized (DI) water and rinsed with ethanol. Zinc sheet metal was purchased from Sigma-Aldrich and cut into strips with 2 mm width and polished before use.

### WO_3_ electrodeposition

All electrochemical depositions and measurements were conducted with an electrochemical workstation (Zahner Zennium CIMPS-1). The deposition of tungsten trioxide on ITO on PET or glass substrates was conducted at −0.3 V in a three-electrode configuration consisting of a Pt wire as counter electrode, Ag/AgCl as the reference electrode and ITO substrates as working electrode. Deposition occurred over five minutes, followed by rinsing of the substrates with DI water. The primer solution was prepared as described elsewhere.^42^ Briefly, 1.8 g of tungsten powder were dissolved in 60 ml of H_2_O_2_ and stirred for two weeks to ensure full dissociation of H_2_O_2_. The obtained solution was filtered to remove any sediments. Afterwards, 0.1 M of HCl was added.

### Hydrogel preparation

All PAM hydrogels were prepared by dissolving respective amounts of potassium persulfate as the initiator in water/DMSO mixtures (10/25/150 mg per 10 ml) followed by stirring at 45 °C for 30 minutes. Subsequently, 1.5 g of acrylamide monomer were added and stirred for 5 minutes. Crosslinking BAM was added and stirred for 10 minutes (10 mg per 10 ml). The resultant transparent solution was poured into a 100 mm polystyrene Petri dish at and kept at 60 °C. Depending on the amount of initiator, polymerization of the hydrogel is completed within 30 minutes. Next, the hydrogels were washed in DI water and soaked in an aqueous solution containing 1 mol l^−1^ ZnSO_4_ and 1 mol l^−1^ AlCl_3_ (ZnAl electrolyte) for 24 hours.

### Assembly of the dual-ion Zn^2+^/Al^3+^ electrochromic device

The electrochromic device was assembled by placing the wet hydrogel electrolyte onto one WO_3_ electrode. The hydrogel was gradually placed onto the electrode to prevent bubbles and ensure good surface contact. Next, a second WO_3_ electrode was brought into contact with the hydrogel. After a 10 minutes of a drying in ambient atmosphere, the hydrogel adheres to the WO_3_ electrode. The hydrogel was cut into shape with a razor blade, leaving a small rim around the WO_3_ electrodes to place the zinc counter electrode.

### Electrochemical measurements and material characterization

All electrochemical measurements including cyclic voltammetry (CV), electrochemical impedance spectroscopy (EIS), and battery cycling (BC) were conducted with an electrochemical workstation (Zahner Zennium CIMPS-1). For EIS measurements, the hydrogel samples were sandwiched between two stainless steel sheets with an effect size of 2 cm^2^. Temperature dependent analysis was conducted in a home-made thermally insulated chamber having real time temperature monitoring *via* a thermocouple. Scanning electron microscopy (Zeiss EVO) was used to analyze morphological properties of the freeze-dried hydrogels. The samples were freeze-dried using a Savant freeze dryer and coated with 7.5 nm of gold. Electron dispersive X-ray (EDX) measurements were conducted during scanning electron microscopy (SEM) imaging to determine composition of the hydrogels. Fourier-transform infrared spectroscopy of dry hydrogels was carried out between 400 and 4000 cm^−1^ to further identify compositional changes (FTIR iS-50 in ATR mode). Field-emission scanning electron microscopy (FE-SEM) was used to examine morphological properties of the electrode materials before and after cycling (Zeiss Sigma FE-SEM). X-ray photoemission spectroscopy (XPS) analysis was conducted on a Kratos AXIS Ultra XPS spectrometer and all analysis was referenced against the C 1s peak at 284.8 eV.

Absorption spectra of the materials and the devices were measured using an UV/Vis-spectrometer (OceanOptics4000). Rise and fall times of the electrochromic switching were determined with a helium–neon (HeNe)-laser and a UDT silicon photodiode.

## Results

**Fig. 1:**
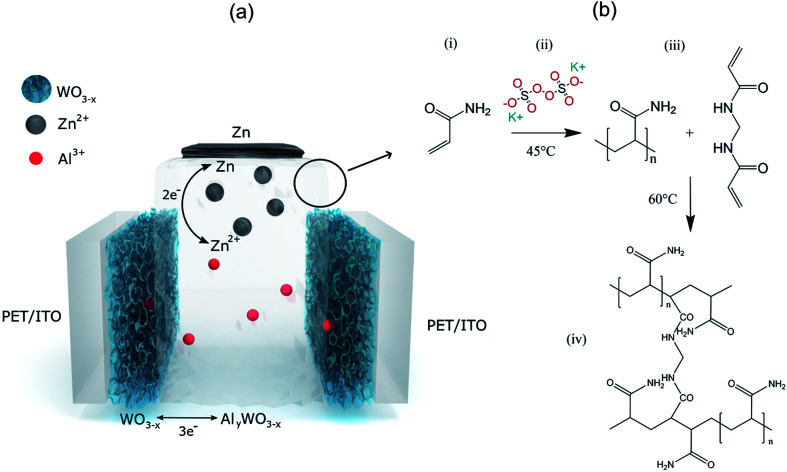
(a) Schematic of the proposed double layer device with Al^3+^ intercalation at the WO_3_ cathode and Zn/Zn^2+^ redox reaction at the zinc anode. (b) General synthesis procedure of polyacrylamide from acrylamide with potassium persulfate as the initiator and *N*,*N*′-methylenebisacrylamide as the cross linker. (i) Acrylamide monomer, (ii) adding acrylamide to a solution of potassium persulfate in DI water kept at 45 °C, (iii) after 5 minutes of stirring at 45 °C addition of BAM and (iv) polymerization of acrylamide with crosslinking at 60 °C.

The basic schematic of the dual-ion rechargeable device is illustrated in Fig. 1(a). Electrochromic tungsten oxide was electrodeposited onto a flexible PET substrate with a layer of 130 nm ITO. Strips of zinc sheet metal served as the anode material. The highly transparent hydrogel forms the electrode separator and prevents short circuiting. Because of the high mechanical flexibility of the PAM-based electrolyte, bending of the device is only limited by the mechanical rigidity of ITO on the respective substrate. By employing two WO_3_ electrochromic electrodes, both the areal capacity and the optical contrast between dark and bleached states are further increased. The small ionic radius of Al^3+^ and its high diffusion coefficient in the hydrogel ensures fast coloration even at a few millimeter electrode distance.^12,43^ Electrodeposition is chosen over chemical methods, because it offers excellent uniformity of surface coverage and potential for large area scalability.^2,44^ To ensure full contact, WO_3_ electrodes were wetted with a thin layer of aqueous mixed ZnAl electrolyte before they were brought into contact with the hydrogel. The conventional polymerization scheme is shown in Fig. 1(b). Sulfate anions act as radical initiator for the polymerization by breaking the vinyl-group double bond of the acrylamide monomer. Polymerization occurs as chain reactions between excited AM monomers, while BAM serves as a crosslinking agent.

**Fig. 2:**
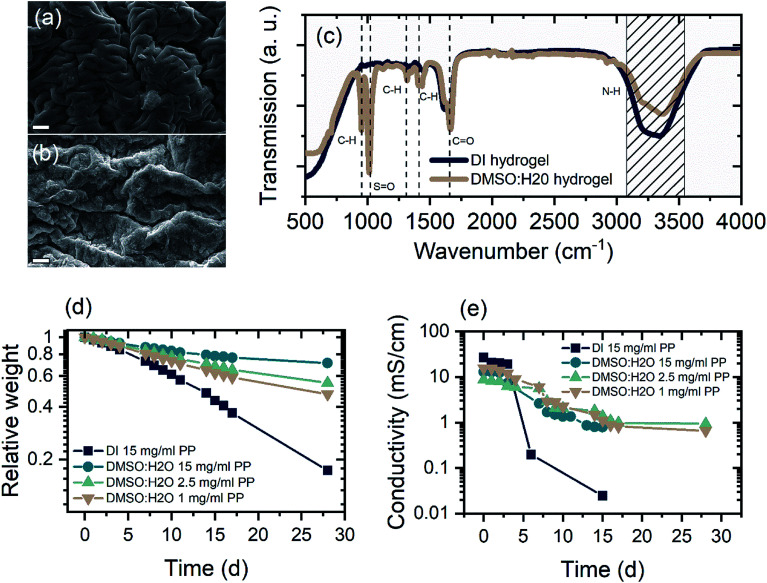
Characteristic properties of the unmodified (DI) and DMSO-modified (DMSO : H_2_O) hydrogel. (a and b) SEM image of freeze dried hydrogels without (a) and with DMSO (b) modification. Scale bar: 2 μm. (c) FTIR analysis of DMSO-modified and DI hydrogel. Important absorption modes are indicated. (d) Relative weight of unmodified and modified hydrogel samples soaked in water for 24 h to ensure full soaking over 30 days at 30% RH. (e) Ionic conductivity over time of DI and DMSO : H_2_O hydrogel samples soaked in ZnAl-electrolyte for 24 h.

The key aspect for a rechargeable device based on hydrogel electrolytes is its lifetime, which is usually limited to a few hours due to electrolyte volatilization. DMSO modification can enhance the electrolyte retention of the hydrogel tremendously. Fig. 2(a) and (b) show a comparison of the morphology of PAM-hydrogel synthesized in pure DI water and in a 50/50 mixture of DMSO : H_2_O. It can be seen from the SEM images that the addition of DMSO to the solvent changes the appearance of the hydrogel. Instead of a “ropy lava”-like lamellar microstructures, DMSO introduces a certain degree of randomization to the polymerization. The formation of these randomized structures can be attributed to reaction-induced phase separation.^45^

FTIR analysis of hydrogels rinsed and soaked in DI water for 24 h shows typical PAM-hydrogel resonances at 3327 and 3185 cm^−1^ for the N–H-stretch and a strong absorption at 1650 cm^−1^ correlated with stretching of C=O bonds (Fig. 2(c)).^46^ On the other hand, the DMSO-modified hydrogel exhibits stronger absorption peaks at 1310 cm^−1^ and 1412 cm^−1^ associated with CH-bending and two additional absorption peaks at 951 cm^−1^ and 1022 cm^−1^. The absorption at 951 cm^−1^ occurs due to the enhanced CH-bending. The newly arising absorption around 1020 cm^−1^ can be attributed to S=O stretching. Furthermore, there is great similarity between the FTIR spectra of the DMSO modified hydrogel and pure DMSO, indicating the incorporation of DMSO molecules.^47^ Electron dispersive X-ray analysis of freeze-dried hydrogels further supports the claim that DMSO is incorporated into the hydrogel microstructure (Fig. S1 and Table S1†). For hydrogels polymerized in mixed solvents, the amount of elemental sulfur increases from approximately 5.6% in the unmodified sample to 13.5% in the 50/50-mixture (Samples were soaked in 1 M ZnSO_4_ for 72 h to ensure to have the same standard). On the contrary, the atomic weight concentration of elemental nitrogen is greatly reduced from 13.6% in the pure DI sample to 3.7% in the DMSO modified sample, suggesting the substitution of the acrylamide's amino group by DMSO. This is also supported by the FTIR where magnitude of the N–H stretch absorption is reduced in the spectra of DMSO modified samples.

In addition to the conventional polymerization process shown in Fig. 1(b), it is possible for the DMSO molecule to polymerize upon interaction with SO^4−^ radicals simultaneously along two pathways.^48^ DMSO can be incorporated into the hydrogel by strong polar interaction or copolymerization (Fig. S2†).^48,49^ The polar S=O group can form hydrogen bonds with water molecules and, thereby, adhere to the hydrophilic amino group (Fig. S2(a)†). As depicted, radical sulfate ions may also interact with DMSO to form dimethyl-sulfone, which in turn polymerizes with AM (Fig. S2(b)†).^48^ As a result, the polar S=O group of the DMSO forms highly stable nanoclusters with the water molecule *via* hydrogen bonds. These nanoclusters significantly alter the physical and chemical properties of the mixed solution^40,47^ and the resulting hydrogel where the S=O bond enhance water retention of the gel, as water molecules are held at sulfoxide sites. Such modifications enable the DMSO-hydrogel to effectively serve as an ion conductor for exceptionally long time spans of weeks even at low temperatures.^45^ For the investigated samples, the ionic conductivity was measured to be 27 mS cm^−1^ and 11 mS cm^−1^ at room temperature and −10 °C, respectively (Fig. S3(c)†).

Long term investigations of the relative weight and the ionic conductivity of unmodified and modified hydrogels show drastically increased retention of the electrolyte incorporated in the modified hydrogel. The relative weight was monitored for samples at 30% RH and the ionic conductivity for samples at 40% RH. This purely to given circumstances of air ventilation in the laboratories. In both cases the relative humidity fluctuated ±2.5%. Fig. 2(d) shows the relative weight of different hydrogel samples over a period of four weeks in 30% relative humidity (RH) atmosphere. Samples synthesized with DMSO show significantly higher water retention, exhibiting between 50% and 72% of its initial weight. Clearly, the concentration of radical initiator influences the water holding capability of the modified hydrogels. Samples polymerized with 1 mg ml^−1^ initiator show 51% weight retention after 4 weeks, while increasing the initiator concentration leads to 60% and 72% weight retention for concentrations of 2.5 mg ml^−1^ and 15 mg ml^−1^, respectively. Even though, decreasing initiator concentration leads to a decrease in water retention of DMSO samples, the experiment shows that the concentration of initiator plays a minor role in the retention of electrolyte. The initiator can crosslink the acrylamide chains, slightly enhancing the specific surface area. This, in turn, enhances the water holding capability of hydrogels^50^ but does not affect the weight retention of hydrogels in a way the polar S=O interaction does. This is evident as the unmodified hydrogel sample polymerized with 15 mg ml^−1^ initiator shows poor weight retention; as a result, the rest weight of the pure DI sample after 28 days is that of its polymeric components.

To investigate the ionic conductivity over time, samples were soaked in mixed electrolytes for 72 h and assembled between 2 cm × 2 cm stainless steel electrodes. To ensure that excess electrolyte evaporated, the first measurements were taken 3 h after assembly. The ionic conductivity was determined as the low frequency intercept of the resulting Cole–Cole-plot. As shown in Fig. 2(e), gel-electrolytes fabricated with DMSO exhibited ionic conductivity as high as 20 mS cm^−1^ after the first 3 h and retained an ionic conductivity of 1 mS cm^−1^ after four weeks of storage in 40% RH. The measured ionic conductivity is in what has been reported, while a retention of 5% ionic conductivity over a period of four weeks is remarkable when compared to other approaches used for electrolyte retention.^38,46,51^ On the contrary, the unmodified hydrogel dries out quickly, reducing its ionic conductivity to 0.2 mS cm^−1^ in only 5 days.

Fig. S4(a)† shows the retention of weight at different RH values for modified samples with 2.5 mg ml^−1^ initiator and without DMSO modification. The DMSO modified samples retain a bigger percentage of their weight over a period of 10 days. The same trend can be observed for the ionic conductivity retention of these samples. DMSO modified samples at 50% RH retain up to 75% conductivity over a period of 10 days, while unmodified samples lose conductivity generally faster than their modified counterparts (Fig. S4(b)†).

A notable aspect of the hydrogel prepared with 2.5 mg ml^−1^ initiator is its high transparency over the whole visible spectrum (90%), which even enhances the transparency of ITO substrates from around 82% to >95%, when in contact (Fig. S5†). Having a refractive index close to that of water (*i.e. n* = 1.33), the hydrogel reduces scattering of light on the rough ITO surface and effectively enhances the optical transparency. The high liquid retention of the gel allows it to be highly transparent even after four weeks of storage (Fig. S6†) at ambient condition. Together with its high optical transparency, long lasting conductivity (*e.g.* 1 mS cm^−1^ after 28 days), and over 1500% stretchability (Fig. S7(c)†), the hydrogel having 2.5 mg ml^−1^ initiator concentration was chosen as the electrolyte matrix for the rechargeable dual-ion device.

**Fig. 3:**
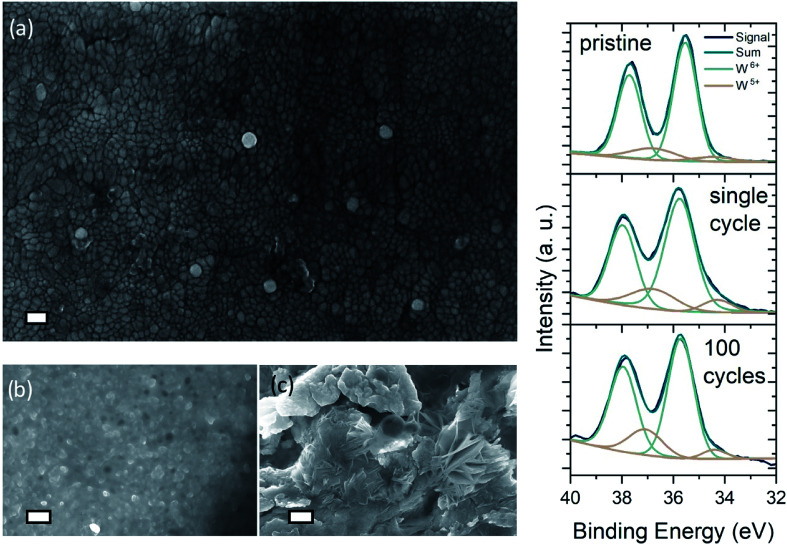
FE-SEM images of neat and cycled electrodes: (a) image of neat WO_3_-electrode with scale bar 100 nm, (b and c) zinc electrode cycled at 200 μA cm^−2^ with DMSO-modified hydrogel (b) and in liquid ZnAl-electrolyte (c) respectively (scale bar = 200 nm). (d) XPS signal of pristine, single-cycle colored and 100 times cycled, aged WO_3_ electrode that has been bleached after 100 cycles. W^6+^ and oxidized W^5+^-states indicated.

Dendritic growth is a major issue associated with cyclic stability of metal anodes.^52,53^Fig. 3(a) shows the FE-SEM image of an as deposited WO_3_ surface on ITO/glass. The nanogranular character of the amorphous tungsten trioxide is apparent, showing grainsizes typically smaller than 60 nm. While it has been reported previously that dendrite formation is prevented when using hydrogel electrolytes,^34^ it is important to examine the effect of dendrite formation when employing the DMSO-modified hydrogel. Fig. 3(b and c) show cycled zinc electrodes in aqueous solutions of 1 M ZnSO_4_/1 M AlCl_3_ and the cycled zinc electrodes with the DMSO-modified hydrogel. Both electrodes were cycled 100 times between 0.4 and 1.2 V *vs.* Zn/Zn^2+^ 200 μA cm^−2^ charge–discharge current with WO_3_ as the counter electrode. From Fig. 3(b and c) it is evident that cycling the zinc electrodes with the DMSO-modified hydrogel suppresses the dendritic growth of Zn(OH)_2_ anode. While dendritic growth can be prevented by utilizing the DMSO-modified hydrogel, cycling of the WO_3_ on ITO/glass electrode in 1 M AlCl_3_ electrolyte shows a retention of 35% initial performance over the course of 300 cycles, exhibiting a specific capacity of 145.8 mF cm^−2^ for the as synthesized electrode and 56.2 mF cm^−2^ for the cycled electrode (Fig. S3(b)†). Such cyclability is much higher has what been reported for Al^3+^ intercalation into V_2_O_5_.^43^

Fig. 3(d) presents the XPS spectra of pristine and a single-cycle intercalated (*i.e.* colored) WO_3_ electrode, as well as a 100 times cycled electrode left in the de-intercalated state. Comparison of the spectra for pristine and colored sample shows increasing emission from surface near W^5+^ states, that can be attributed to reduction of tungsten by Al^3+^-intercalation. The area of the W^5+^ peaks decreases just slightly for the aged sample, which indicates that de-intercalation after 100 cycles does not restore the sample to its pristine state. Analysis of the O 1s spectra shows an indication of irreversible intercalation at the surface. As the density of O^2−^ lattice sites decrease, a greater amount of oxidized oxygen states arises in the 100 times cycled sample (Fig. S9(b)†). Furthermore, the XPS analysis (*i.e.* the onset of an aluminum peak in Fig. S9(a)†) confirms that aluminum intercalates into the surface of the WO_3_ layer. Nevertheless, the necessity of Al^3+^-ions in the DMSO-modified hydrogel electrolyte becomes obvious when examining at the ionic impedance of the DMSO-modified hydrogels with 1 M ZnSO_4_ and with 1 M ZnAl (Fig. S3(a and c)†). The mixed ZnAl electrolyte shows an extremely higher ionic conductivity of 20 mS cm^−1^, while the same hydrogel in 1 M ZnSO_4_ exhibits conductivity of only 5 mS cm^−1^.

In mixed electrolytes, concerns about each species' contribution to the device performance arise due to the possibility of intercalation of the ion's hydrated form. Moreover, precipitation and salt scale build-up are possible. The mechanism of aluminum ion intercalation is still an ongoing topic in battery and electrochromic device research. Whether or not the bare ions intercalate or if they do so in their hydrated form, is being discussed widely, but would not change the underlying principle of this device. Bare Al^3+^ ions have a significantly smaller radius than Zn^2+^ ions (Zn^2+^ (0.74 Å) > Al^3+^ (0.53 Å)), which holds true for their hydrated forms as well (Zn^2+^ (4.3 Å) > Al^3+^ (1.90 Å)).^54,55^ XPS analysis of a cycled WO_3_ shows clearly that aluminum intercalates into the WO_3_ electrode (Fig. S9†). Furthermore, EIS measurements of the hydrogel's conductivity show clear favoritism for Al^3+^ conduction. Through a series of extensive measurements it was shown that in the mixed electrolyte Al^3+^ ions are favored to intercalate into WO_3_.^56^ Here, EDX analysis of a device at different cycles of a life span shows no indication for species build up at the surface (Table S2†). EDX analysis of an electrochromic device cycled for one time and 2000 times (postmortem) shows no indication for salt scale formation or buildup of other precipices, as the atomic concentration relation of Zn to Al stays unchanged. Furthermore, the amount of Zn is 5 times lower on and in the sample, suggesting low amounts of Zn^2+^ intercalation, while Al^3+^ can intercalate easily.

**Fig. 4:**
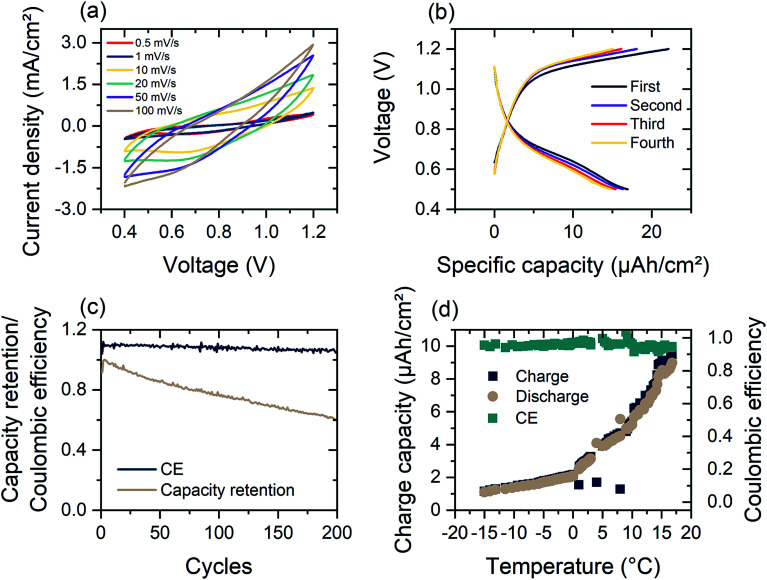
(a) Cyclic voltammetry measurement of the electrochromic dual-ion device assembled with DMSO-modified hydrogel electrolyte between 0.4 V and 1.2 V *vs.* Zn/Zn^2+^ at different cycling speeds, (b) first four charge–discharge profiles of dual-ion device under constant current of 10 mA cm^−2^, (c) capacity retention and coulombic efficiency (CE) over 200 cycles of charge–discharge at 50 mA cm^−2^, (d) temperature dependence of charge–discharge capacity and CE of electrochromic device with DMSO-modified hydrogel electrolyte.

As shown in Fig. 4(a), the dual-ion WO_3_-zinc device, assembled on a flexible ITO/PET substrate, exhibits high faradaic capacitances of 122.9 mF/cm^2^for a sweeping speed of 100 mV s^−1^ and up to 375 mF cm^−2^ for 0.5 mV s^−1^ (referenced against area of WO_3_ electrode). The faradaic capacitance was determined using the equation:
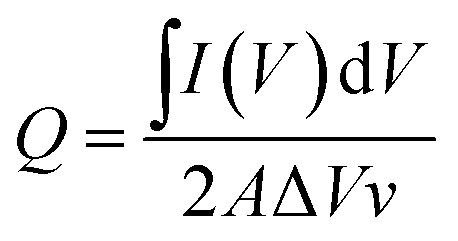
where, *I*(*V*) is the measured current a voltage (V), *A* is the electrode area, Δ*V* is the voltage potential window, and *v* is the sweeping rate. Slower cycling allows for efficient Al^3+^ ion diffusion into the WO_3_ layer, which in return, leads to intercalation of Al^3+^-ions at deeper lying oxygen vacancies. Moreover, since the total area enclosed by the oxidation/reduction curves are similar, intercalation and stripping processes occur reversibly between 0.4 V and 1.2 V. The expected reduction/oxidation reactions would follow:Zn_(aq)_^2+^ + 2e^−^ → Zn_(s)_ at *E* = −0.76 VWO_3_ + *x*Al_(aq)_^3+^ + 3*x*e^−^ → Al_*x*_WO_3(s)_ at *E* = 0.34 V

Following the theoretical redox potentials, a maximum cell voltage of 1.1 V should be possible. As can be seen in Fig. S7,† the Al intercalation into WO_3_ creates a distinct peak at 0.65 V, while the zinc redox reaction occurs steadily. The lower potential window is mainly due to irreversible effects occurring when increasing the potential window. The resistance of the hydrogel electrolyte leads to diffusion-based voltage drops, leading to increased stripping of the Zn electrode.

The CV curves can further be analyzed towards ion insertion or diffusive contribution dependent on the square root of the sweep rate and capacitive sources of the overall capacity (*q*_c_).^57^ Based on the following equation, the capacitive part of the energy storage can be determined as the *y*-intercept of the plot of the capacity over *v*^−1/2^ (Fig. S10†).*q*_tot_ = *q*_c_ + const(*v*^1/2^)

The diffusive contribution towards the overall capacity increases from 22% for a scan rate of 100 mV s^−1^ to 63% for 1 mV s^−1^ scan rate due to the resistance of the hydrogel electrolyte.

Fig. 4(b) shows the galvanostatic measurement of the flexible device. The observed intercalation peak at 0.65 V is visible as a shoulder in the discharge current. In constant current charge–discharge measurements at high current densities of 200 μA cm^−2^, the dual-ion device has areal discharge capacities of up to 16.9 μA h cm^−2^ However, at lower current densities (<200 μA cm^−2^), the initial charge–discharge cycles exhibit poor reversibility (Fig. S3(d)†). This is mainly due to irreversible stripping of zinc at the anode during discharge. Zn^2+^ ions go into solution and do not get plated during charging. When increasing the current density to 0.4 and 1 mA cm^−2^ the discharge capacity reduces to 14.2 and 9.3 μA h cm^−2^ respectively (Fig. S11†). The coulombic efficiency increases for higher charging currents, resulting from shorter time spans for irreversible zinc stripping. Faster cycling therefore favors reversibility, at the expense of less capacity.

As depicted in Fig. 4(c), the coulombic efficiency (CE) of the dual-ion device remains constant for over 200 cycles of charging and shows a high retention of 60% of the initial discharge capacity. The drop in the charge capacity is attributed to the irreversible intercalation of Al^3+^-ions, which results in a change of the open circuit potential from 1.05 V to 0.8 V. Intercalation of Al^3+^ into the WO_3_ electrode further leads to reduced series resistance for the dual-ion device (Fig. S3(a)†). This results in turn to an insulator–metal transition in WO_3_ that further enhances the electrical conductivity of the electrode.^58,59^ The limiting factor of device capacity is Zn electrode, which is relatively new as an anode material in electrochromic devices and dual-ion batteries, while WO_3_ has been investigated for years. The interplay of zinc and its respective counter electrodes requires further investigation. Furthermore, the limited ionic conductivity of the hydrogel electrolyte will prevent the cell from reaching its full potential, but nevertheless providing improved functionality.

Fig. S12† illustrates the device's cycling performance at various hydrogel electrolyte concentrations. The highest reversible charge capacity of 17.5 μA h cm^−2^ is obtained for an equal concentration of the Al^3+^ and the Zn^2+^ ions. Furthermore, the coulombic efficiency stays between 0.92 and 0.95. By increasing the concentration of Al^3+^ ions, the first cycles exhibit high irreversible discharge which is attributed to Zn stripping from the Zn anode. The coulombic efficiency of the Al^3+^ rich electrolyte does not exceed 0.7. At high concentrations of Zn^2+^ ions the coulombic efficiency is between 0.9 and 0.95 in the first ten cycles, but the overall capacity is reduced, due to a higher barrier for intercalation into the WO_3_ electrode. Clearly, the optimum charge–discharge cycling is achieved close to the balanced 1 : 1 ratio of the ions. These findings are further supported by elemental analysis of a hydrogel sample cycled in an electrochromic device (Table S3†). Here, after cycling, an elemental composition ratio of Al^3+^ to Zn^2+^ is found to be 1.2 : 1 (with 1.16 : 1 for the pristine soaked hydrogel). The equilibrium concentration of the system lies at such concentrations.

The modification of the hydrogel allows for operation in a large temperature range. While commercial Li-ion batteries should not be operated at temperatures below 5 °C, the electrochromic Zn^2+^/Al^3+^-device is able to deliver a specific capacity of 1 μA h cm^−2^ at −15 °C at a charging current of 1 mA cm^−2^ (Fig. 4(d)). As shown in Fig. 4(d), the low temperature (−15 °C) capacity presents a performance of around 10% of room temperature operation. The DMSO-modified hydrogel electrolyte consequently shows an increased ionic impedance at −10 °C (Fig. S3(c)†). The DMSO modification of the hydrogel also leads to increased device life time and retains 20% initial discharge capacity after 10 days of storage at 60% RH and 22 °C (Fig. S8(d)†).

**Fig. 5:**
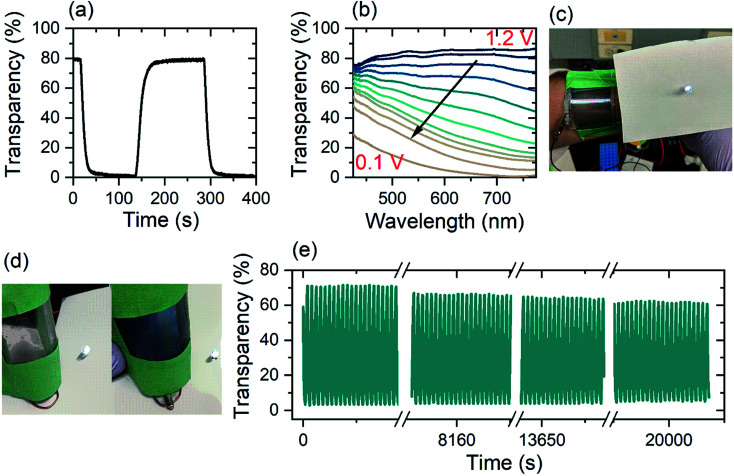
(a) Rise and fall of colored-bleached cycling of electrochromic double layer device with DMSO-modified electrolyte between 0.1 V and 1.2 V, measured at 632 nm wavelength. (b) Transmission spectra of single layer device with DMSO-modified electrolyte, (c and d) photo of bendable device as wearable device with zinc electrode on the stuck out side of the device (e) colored-bleached cycling over 1000 cycles with a switching time of 22 s between 0.1 and 1.2 V. The measurements were done at a wavelength of 632 nm.

Energy storage devices based on electrochromic materials function as supercapacitors on the basis ion intercalation into TMO electrode.^60,61^ Herein, the electrochromic double-layer dual-ion device investigated possesses a high optical transparency in the charged state (80%) and a low optical transparency when discharged (0.5%) (Fig. 5(a)). Over the whole visible spectrum (425–775 nm), a single-layer (*i.e.* one WO_3_ electrode) Zn^2+^/Al^3+^-device has a transparency of over 80% in its charged state. During discharge from 1.2 V to 0.1 V, light absorption shifts from the near-infrared region and across the whole visible spectrum. In the fully discharged state (0.1 V) the single layer device has a transparency of 5% at a wavelength of 632 nm (Fig. 5(b)).

Fig. 5(a) shows the time-resolved coloration/bleaching of the double layer, flexible Zn^2+^/Al^3+^-device for a wavelength of 632 nm (Fig. 5(c and d)). By employing the double layer configuration, the optical contrast is enhanced. While the maximum transparency of the two different configurations is nearly unchanged, the double layer structure has an opacity of 99.5% of incident light (Fig. S8(e)†) whereas, the single layer device blocks 95% of the light (Fig. 5(b)). In both arrangements, the coloration efficiency is found to be 54 cm^2^ C^−1^. Stability measurements of the dual-ion device show high retention of contrast over 1000 cycles or 22 500 s (Fig. 5(e)) at a wavelength of 632 nm, indicating that irreversible surface intercalation does not prevent efficient device performance. While the transmission in the colored state remains mainly constant for the whole window of investigation, the transparency in the bleached state reduces by 10%. This reduction in the optical transparency can be explained by irreversible Al^3+^-intercalation into the WO_3_,^26^ which is supported by EDX analysis of a device after its life cycle (Table S2†). This decay can be eliminated *via* a galvanostatic treatment.^62^

The electrochromic device is highly flexible while retaining both its electronic and optical properties. In order to make it a suitable wearable device and wrap it around the forearm for example, the bending curvature needs to be 180° at a radius of 2 to 3 cm (compare Fig. 5(c)). For the wearable device, the device configuration was altered and is depicted in Fig. S13.† By changing the position of the zinc electrode, skin contact can be avoided, and therefore any possibly arising unwanted reactions. In a simulated wearing situation, the electrochromic device was repeatedly bent to a radius of curvature of 50 mm prior to measuring its capacity. The device suffered an initial loss of capacity, but remarkably retained 70% of its capacity over 30 cycles of bending (Fig. S8(b)†). The initial loss arises mainly due to cracking of the ITO layer when driven beyond the manufacture's during safe bending curvature of 75 mm. In a similar experiment, when a repeated pressure of 1.5 kPa was applied to the dual-ion electrochromic device, after 30 cycles, the electrical performance was only reduced by 10% (Fig. S8(c)†). In both, the bending and the pressure application experiments, the CE remained basically unaltered. This indicates that the slightly decreased performance is mainly due to the ITO/PET substrate mechanical stability and not due to the electrochemical processes.

## Conclusion & outlook

In conclusion, we modified the water retention properties of a polyacrylamide-based hydrogel by exploiting the microscopic interaction of DMSO : H_2_O nanoclusters. Remarkably, exploitation of cluster formation enables the DMSO-modified hydrogel to show a retention of 5% initial ionic conductivity after four weeks of storage at 30% RH and can be operated at temperatures as low as −15 °C. Ion conduction in the DMSO-modified ZnAl hydrogel-electrolyte shows ionic conductivity up to 27 mS cm^−1^ and provides high flexibility with more than 1500% stretchability. These key features together with its high transparency, make the developed DMSO-modified hydrogel an impeccable ion conductor for the proposed double layer electrochromic dual-ion device. The introduced device is to our knowledge the first quasi solid-state electrochromic device based on zinc and holds the promise of featuring a battery with electrochromic functionality even as wearable, personal electronics. We achieved charge capacities as high as 16.9 μA h cm^−2^ or 375 mF cm^−2^ for the flexible dual-ion device, which can technically be doubled by using a double layer device architecture. The double layer architecture also drastically improves the electrochromic properties of the device, leading to absorption of 99.5% of incident visible and near-IR light. DMSO-modification of the hydrogel allows exceptional device operation over 10 days without encapsulation; the DMSO-modified hydrogel is still not dried out after 90 days of storage.

## Conflicts of interest

The authors declare no conflicts of interest.

## Supplementary Material

RA-009-C9RA06785J-s001
