# Correction: Rechargeable Zn^2+^/Al^3+^ dual-ion electrochromic device with long life time utilizing dimethyl sulfoxide (DMSO)-nanocluster modified hydrogel electrolytes

**DOI:** 10.1039/c9ra90083g

**Published:** 2019-11-13

**Authors:** Eric Hopmann, Haizeng Li, Adulhakem Y. Elezzabi

**Affiliations:** Ultrafast Optics and Nanophotonics Laboratory, Department of Electrical and Computer Engineering, University of Alberta Edmonton Alberta T6G 2V4 Canada hopmann@ualberta.ca

## Abstract

Correction for ‘Rechargeable Zn^2+^/Al^3+^ dual-ion electrochromic device with long life time utilizing dimethyl sulfoxide (DMSO)-nanocluster modified hydrogel electrolytes’ by Hopmann Eric *et al.*, *RSC Adv.*, 2019, **9**, 32047–32057.

The corrected author listing (first/last) names is shown above.

The Royal Society of Chemistry apologises for these errors and any consequent inconvenience to authors and readers.

## Supplementary Material

