# Supplementary material for: Rechargeable Zn2+/Al3+ dual-ion electrochromic device with long life time utilizing dimethyl sulfoxide (DMSO)-nanocluster modified hydrogel electrolytes
Source: RSC Adv. 2019 Oct 9;9(55):32047–57. doi: 10.1039/c9ra06785j (PMC9072954; doi:10.1039/c9ra06785j)
Supplement: RA-009-C9RA06785J-s001 [file RA-009-C9RA06785J-s001.pdf]

Electronic Supplementary Material (ESI) for RSC Advances.
This journal is © The Royal Society of Chemistry 2019

Electronic Supplementary Information

Rechargeable Zn^{2+}/Al^{3+} dual-ion electrochromic device with long life time utilizing Dimethyl sulfoxide (DMSO)-nanocluster modified hydrogel electrolytes

Hopmann Eric*, Haizeng Li*, and Elezzabi Adulhakem Y.

Ultrafast Optics and Nanophotonics Laboratory, Department of Electrical and Computer Engineering, University of Alberta, Edmonton, Alberta, T6G 2V4, Canada

*corresponding authors: hopmann@ualberta.ca, haizeng@ualberta.ca

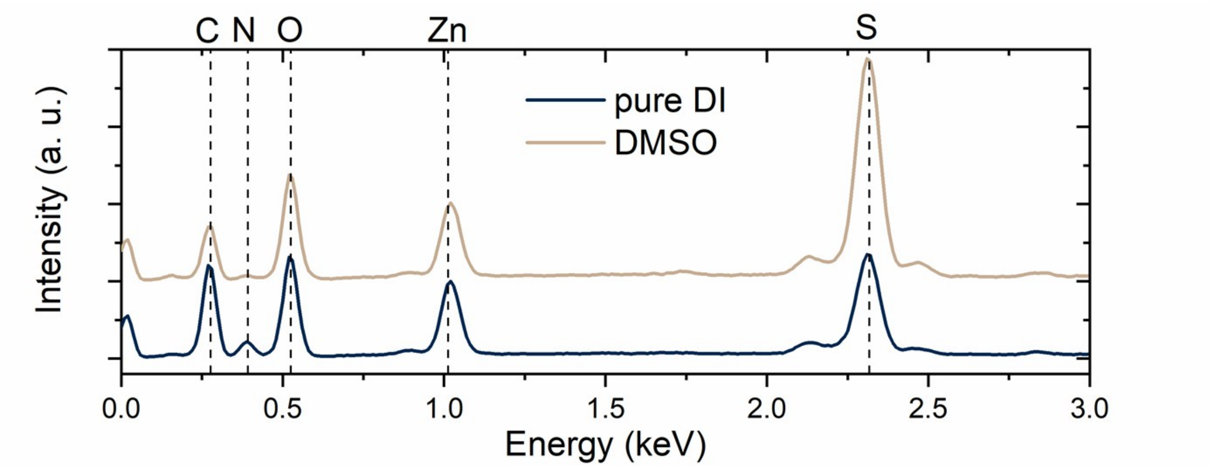

Figure S1: EDX spectra of dried hydrogels with and without DMSO modification.

Fig. S1 shows the EDX spectra of dried hydrogel samples. Both samples were soaked in $ZnSO_4$ for 72h to create a reference background of Zn. Freeze drying was conducted for over 24h to ensure full drying. The sample with DMSO modification shows a stronger signal for S, while the amount of elemental N is greatly reduced. The signals for C and O stay relatively unchanged. The analysis of peak areas gives the elemental composition of the hydrogel. Table S1 lists the weight composition of the hydrogels.

Table S1: Elemental composition of hydrogel samples with and without DMSO modification as determined by EDX

	Pure DI		DMSO modified	
Element	Wt%	Wt% σ	Wt%	Wt% σ
C	39.2	0.34	39.91	0.39
N	13.58	0.44	3.69	0.41
O	31.65	0.29	32.37	0.3
S	5.61	0.06	13.45	0.12
Zn	9.96	0.13	10.57	0.15
Total	100		100	

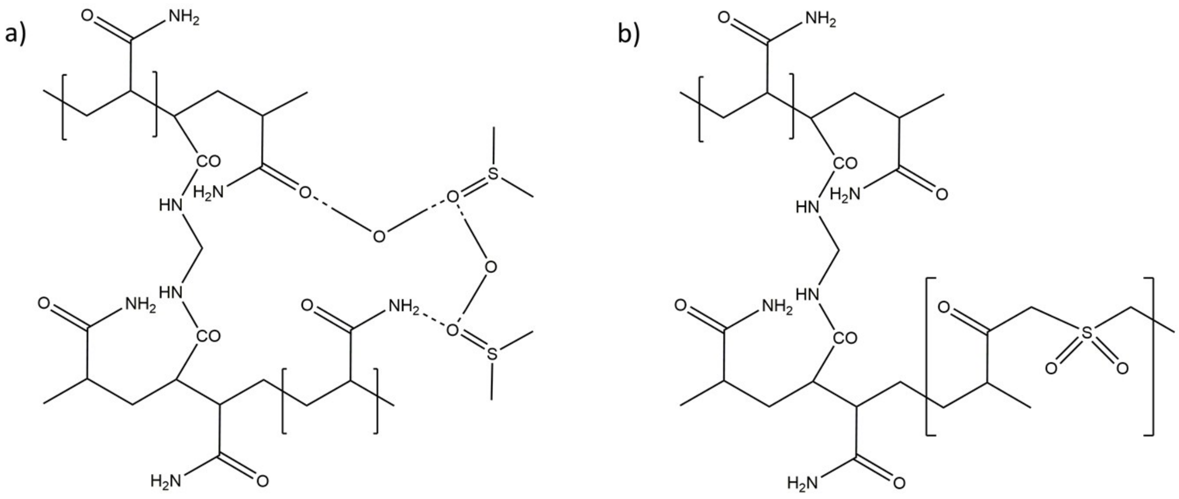

Figure S2: Possible mechanisms of DMSO incorporation into PAM-hydrogel. (a) Water molecule mediated dipolar interactions. (b) Copolymerization of sulfone with acrylamide.

Fig. S2 shows the proposed mechanisms for DMSO incorporation upon gelation of acrylamide. The acrylamide is still crosslinked by BAM. DMSO can be incorporated by dipole-dipole-interaction of the highly polar S=O group of the DMSO with hydrogen of water molecules (a). Furthermore, it was suggested that DMSO might be radicalized by sulfate ions, which might lead to copolymerization of DMSO and acrylamide (b) [1].

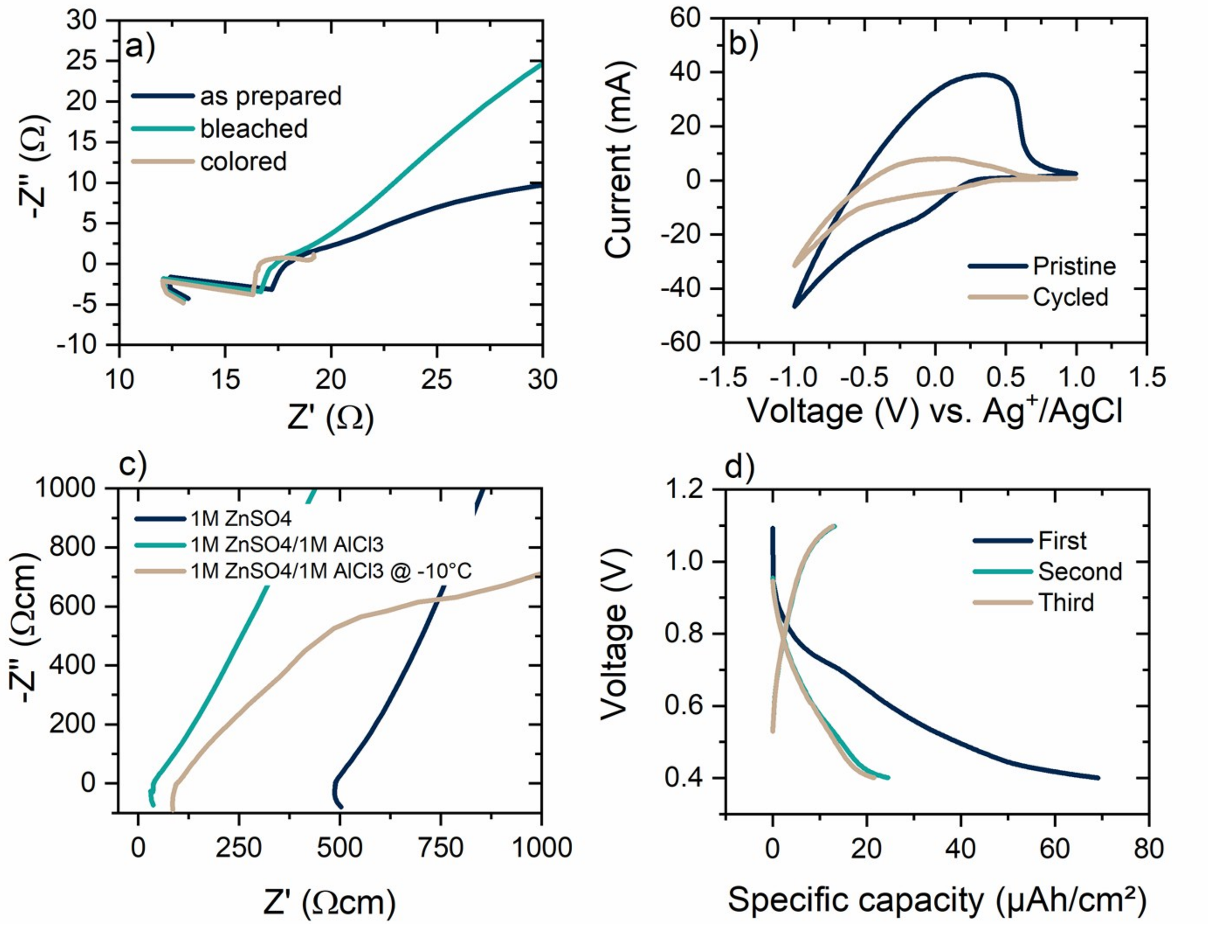

Figure S3: Electrochemical properties of the electrolyte, electrodes and device. (a) EIS analysis of the dual ion electrochromic device during cycling. (b) Cyclic voltammetry at first cycle and 100 cycles of WO_3 electrode at a sweep rate of 100 mV/s. (c) EIS measurement of hydrogel with different electrolyte solutions. (d) Charge-discharge profiles of dual-ion device at 5 mA/cm² (i.e. device initially in charged state).

In Fig. S3(a), an assembled single layer dual-ion device on ITO/glass was tested towards its ionic conductivity in operation. The fresh device shows a resistance of 17 Ohm as the x-intercept, which is reduced to 16 Ohm when colored and 16.5 Ohm for the bleached device, indicating the insulator/metal transition of the device [2]. Resistance values were determined as the high frequency intercept with the x-axis following a Randles Cell model for an equivalent circuit with mixed kinetic and charge-transfer control (Fig. S14).

Fig. S3(b) shows CV cycling of a first and a 100 times cycled device with a sweep rate of 100 mV/s showing strong decrease in retention. Fig. S3(c) shows the ionic resistivity of the hydrogel with 2.5 mg/ml initiator concentration and DMSO modification. The ionic conductivity of hydrogel with pure $ZnSO_4$ is around 2 mS/cm, increasing to 25 mS/cm for the mixed electrolyte. The mixed electrolyte retains a conductivity of 11 mS/cm at -10°C. Fig. S3(d) shows the initial charge/discharge cycles of an assembled device at 100 μA/cm^2.

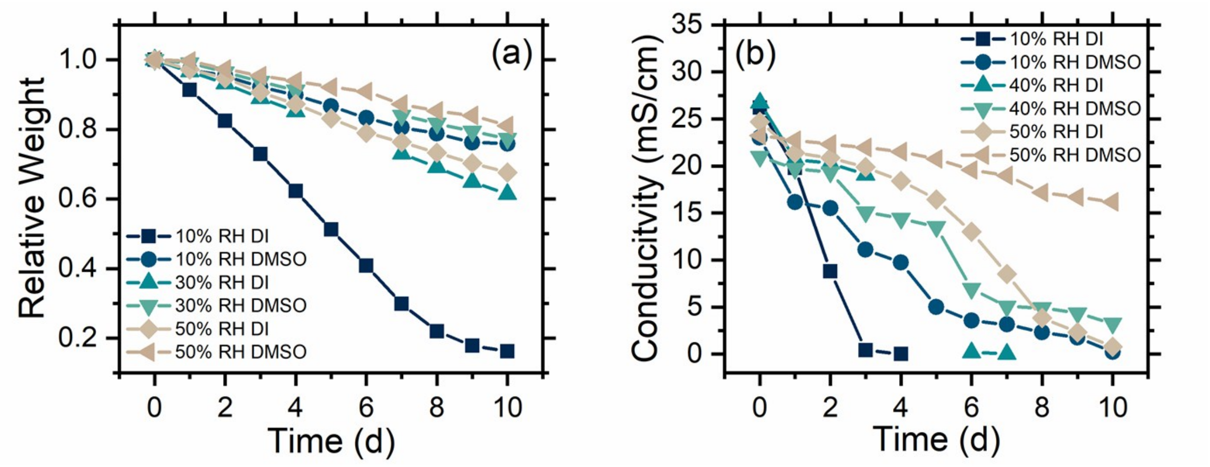

Figure S4: (a) Relative Weight and (b) ionic conductivity over time for samples with and without DMSO modification over time. The DMSO modified sample has an initiator concentration of 2.5 mg/ml

Fig. S4 shows the relative weight and ionic conductivity of hydrogel electrolyte samples over a period of 10 days. The DMSO modified sample contains 2.5 mg/ml initiator. For a relative humidity of 10% the unmodified sample dries out after approximately 8 days and does not show any conductivity after 4 days. The modified sample retains 5% of its conductivity after 10 days. With increasing humidity, the unmodified samples retain their conductivity longer, but not as long as the modified samples. The DMSO modified hydrogel retains nearly 75% ionic conductivity after 10 days of storage at 50% RH. The RH was held constant by nitrogen and air flow into a closed of box.

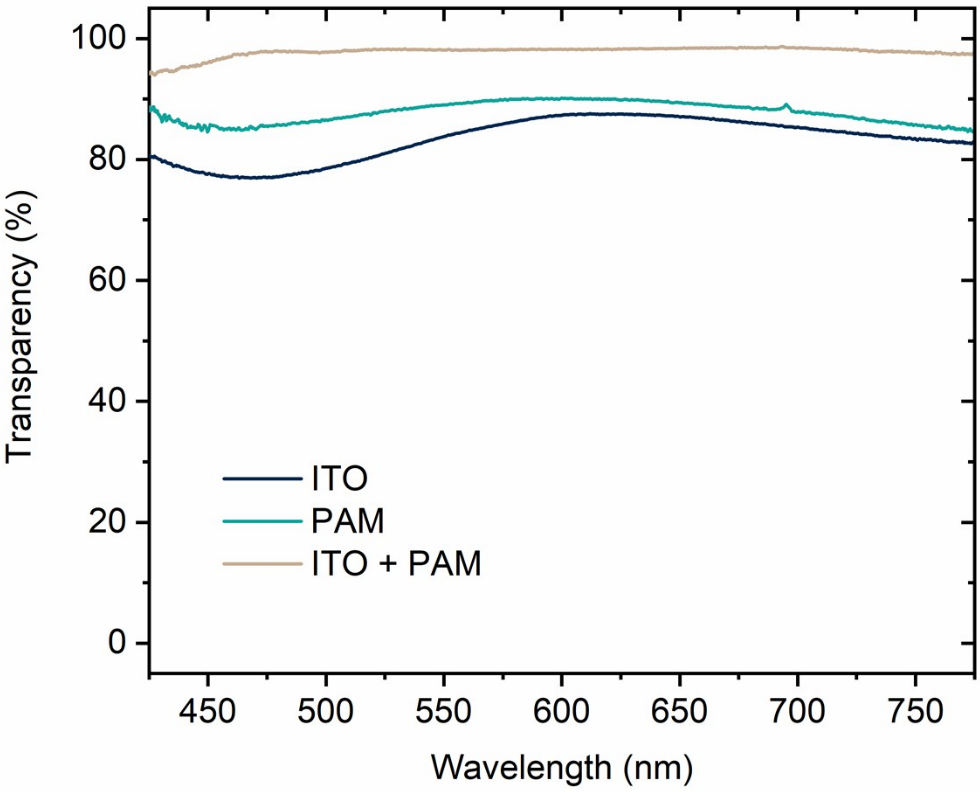

Figure S5: Optical transparency of ITO/PET substrate, DMSO-modified hydrogel and a combined ITO/PET/DMSO modified-hydrogel.

Optical transmission spectra of ITO/PET, the polyacrylamide hydrogel and the combination of both. The optical transmission of the single substrate is around 82% throughout the visible spectrum. PAM shows slightly increased transmission around 90%, while the combination of PAM and ITO shows nearly perfect transmission around 95%. The increase in transmission can be attributed to the change at the ITO interface. When in contact with PAM, the scattering from the ITO surface roughness is decreased.

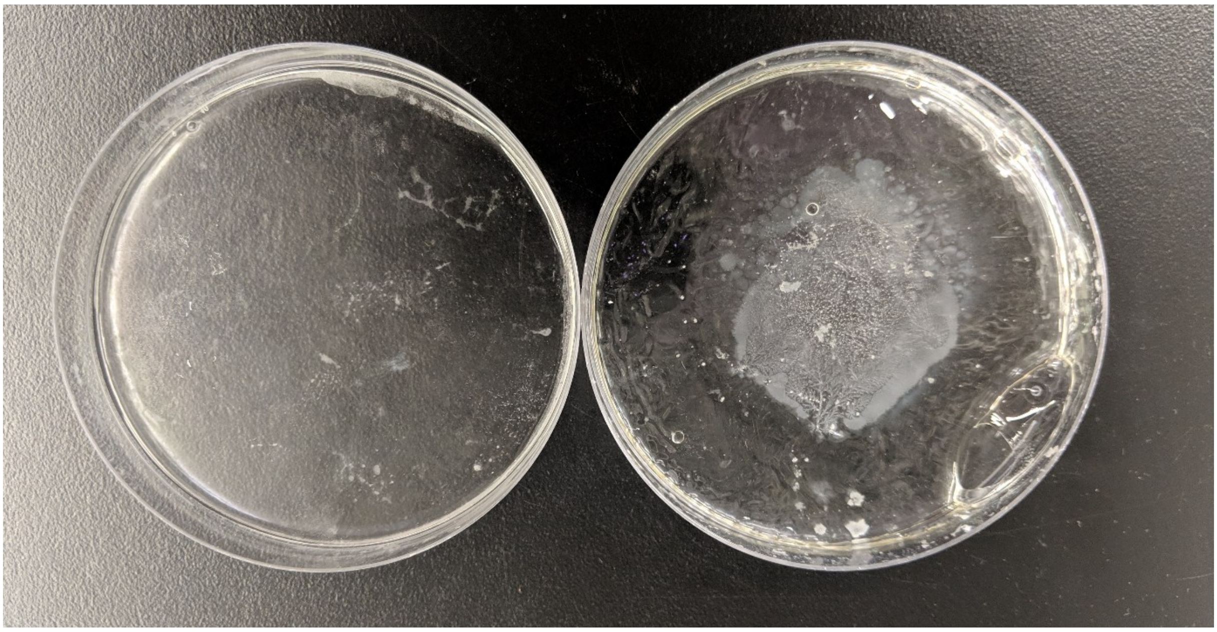

Figure S6: Photographs of hydrogel samples after 28 days of drying with (left) and without (right) DMSO modification at 40% RH.

Fig. S6 depicts a photograph of two hydrogel samples with and without DMSO modification, which have been dried under 40% RH for 4 weeks. The drying process leads to crystallization of the electrolyte, which transforms the hydrogel to become translucent. While this happens for the unmodified sample, the DMSO modified sample is still flexible and most importantly it remains highly transparent.

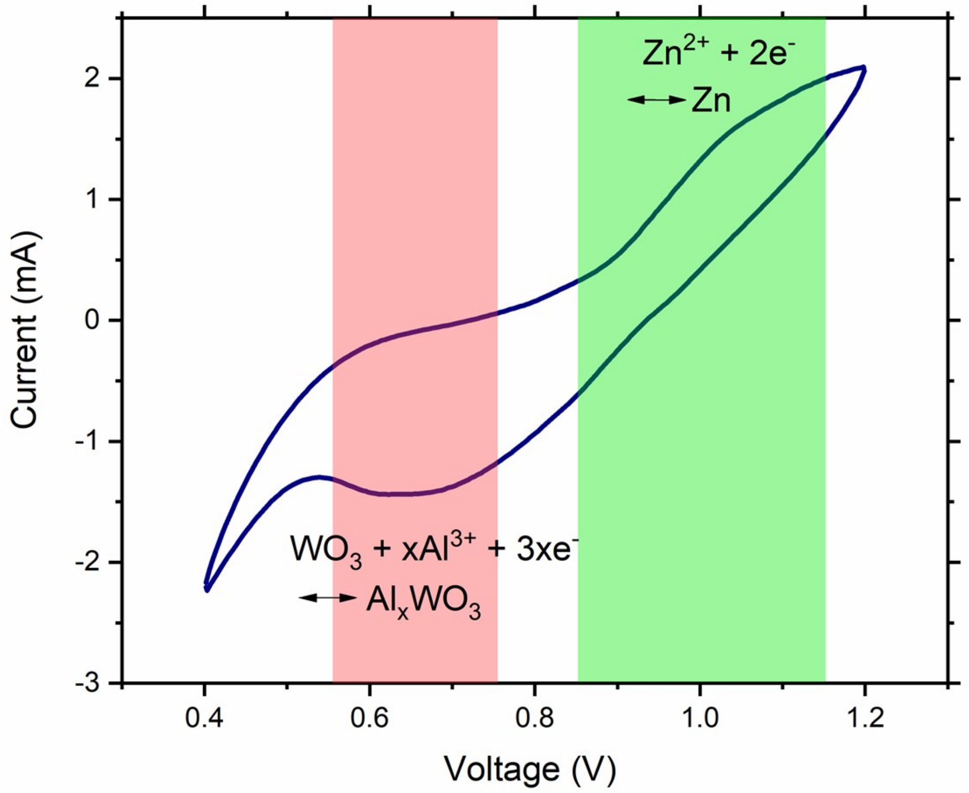

Figure S7: CV measurement of WO3-zinc electrochromic device at 0.5 mV/s sweep rate. Sample was cycles with mixed electrolyte hydrogel.

Fig. S7 illustrates the cyclic voltammetry of the electrochromic device at 0.5 mV/s. Indicated in red and green are the redox reaction peaks that can be observed in the plot. The peak at 0.65 V can be attributed to Al-intercalation into WO_3. Deintercalation cannot be attributed to a single peak and rather occurs over a broad range of voltage values (0.55 V to 0.75 V). The Zn redox reaction occurs steadily over 0.8 V.

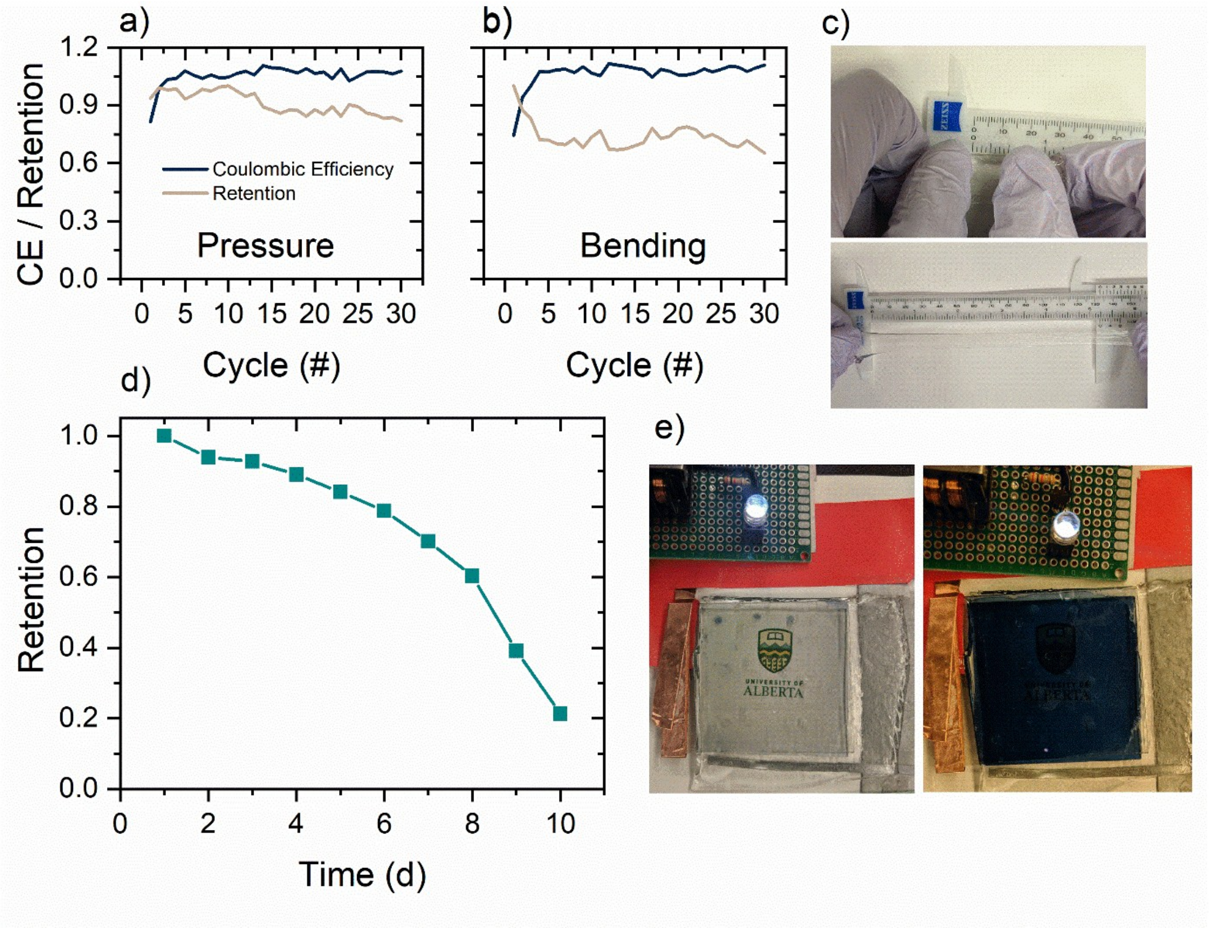

Figure S8: Retention of device capacity over 30 cycles of repeated pressure (a) and bending (b). (c) Images of unstretched and stretched hydrogels (c). (d) Retention of the dual-ion device capacity at 60% RH over an investigation period of 10 days (J = 20 mA/cm ^{2}). (e) A photograph of charged (bleached) and discharged (colored) states of the electrochromic dual-ion device.

Fig. S8(a,b) show the capacity retention and CE of two assembled double layer dual-ion electrochromic device under repeated bending and pressure. Coulombic efficiency here refers to the discharge capacity divided by charge capacity. The device was bent to a curvature of 50 mm and the repeated pressure was 1.5 kPa. Fig S8(c) shows a photograph of a hydrogel strip of 1 cm width stretched to over 15 cm. Fig. S6(d) shows the retention of discharge capacity of the dual-ion electrochromic device after 10 days of storage at 60% RH. The discharge current was 400 μA/cm ^{2}. Fig. S8(e) shows a photograph of the dual-ion electrochromic device in the bleached and colored state (charged/discharged).

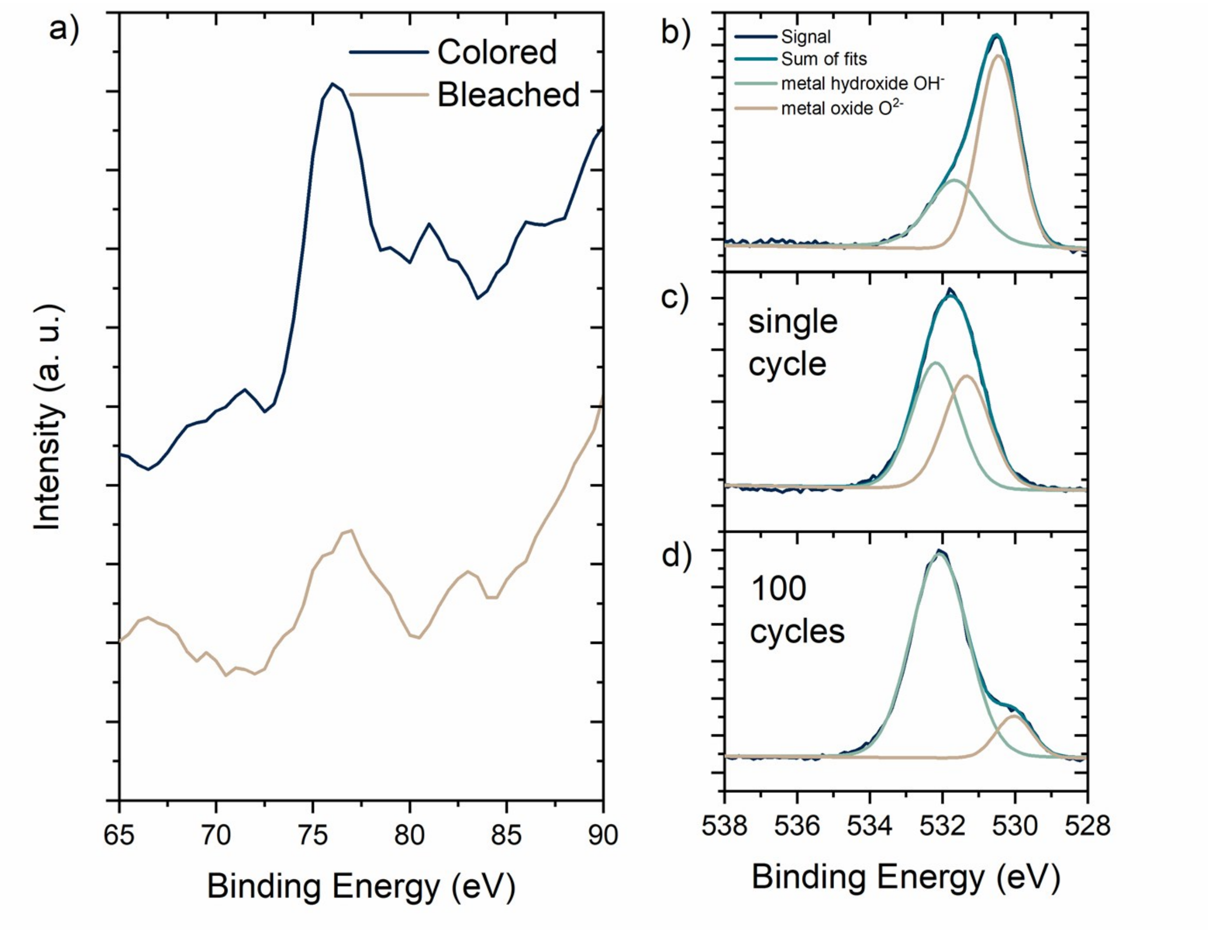

Figure S9: (a) XPS spectra of Al2p-region with early onset of Al2p X-ray emission. XPS spectra of oxygen 1s region corresponding to lattice oxygen O^{2-} (530 eV) and OH^- as defect oxygen (531.9 eV) in (b) Pristine, (c) single cycle, (d) and 100 times cycled WO_3 electrode.

Fig. S9(a) depicts the XPS spectra of a colored and bleached WO_3 sample in the Al2p energy region. The WO_3 electrodes were bleached/colored in aqueous mixed electrolytes before investigation and repeatedly rinsed in DI water to remove traces of the electrolyte. For the colored device, there is a slight increase in aluminum signal, which is a signature of aluminum intercalation.

Fig. S9(b-d) illustrates the XPS signal of O1s in a pristine, single cycled and 100 times cycled WO_3 electrode. The figures indicate that lattice oxygen gets replaced when Al^{3+} intercalation occurs, while the amount of hydrolyzed oxygen increases.

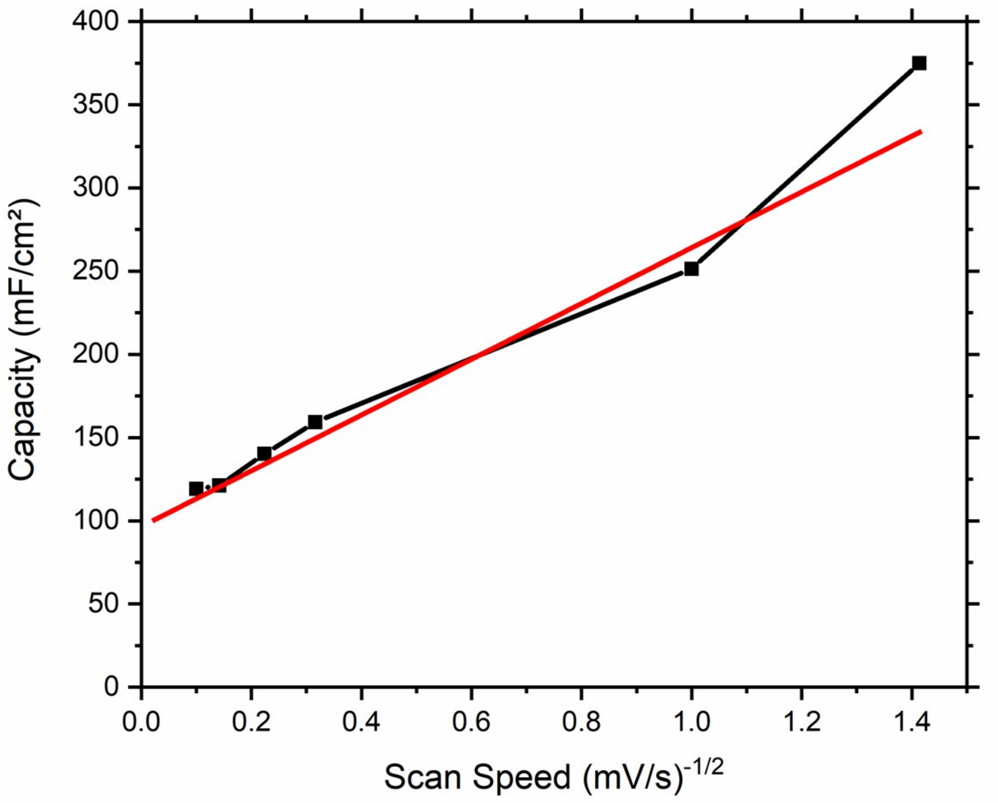

Figure S10: Capacity extracted from the CV-measurements in Fig. 4(a) over one over the square root of the scan speed in mV/s. The red line shows the linear fit of the values. The y-intercept is 96.7 mF/cm².

Fig. S10 shows the faradaic capacity over the square root of the scan rate. By interpolating the experimental values (black), the surface capacitive contribution towards the overall capacity can be determined.[3] The intercept is 96.7 mF/cm², and the slope is 167 mF/cm².

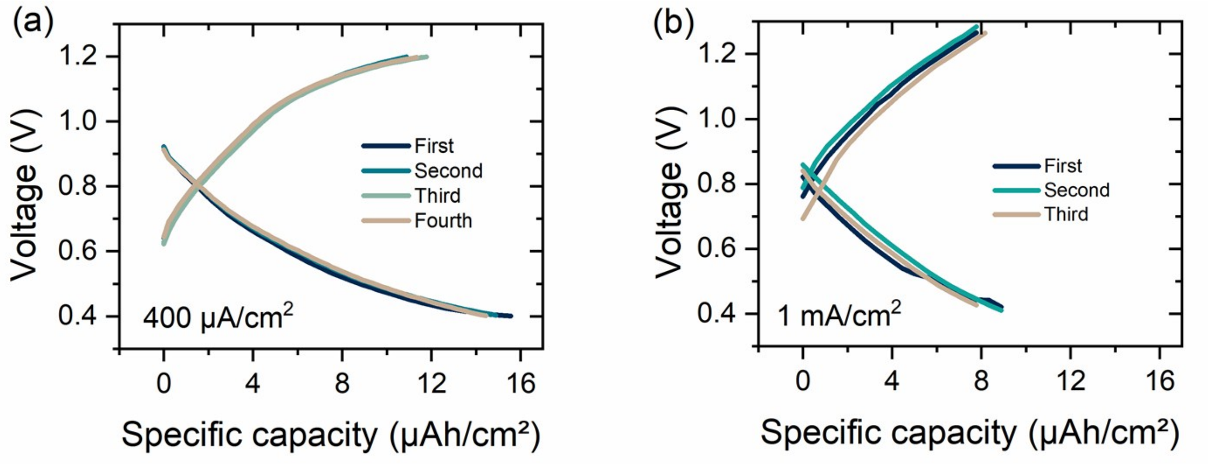

Figure S11: Charge- discharge profiles for electrochromic dual-ion batteries at: (a) 400 μA/cm² and (b) 1 mA/cm² current density.

Fig. S11(a) and (b) show charge discharge profiles for electrochromic double layer dual-ion batteries on ITO/PET. The 1 mA/cm² cycled device has a wider potential window between 0.4 and 1.3 V. In the case of (a), the discharge capacity is slightly higher than the charge capacity, indicating irreversible stripping of Zn.

Table S2: EDX analysis of a pristine, one time cycled and post mortem (1000 cycles) WO_3 electrode of an electrochromic device in ZnAl electrolyte. EDX analysis was conducted at 20 kV at 2000 times magnification.

Pristine		First cycle		Post Mortem	
Element	Wt%	Element	Wt%	Element	Wt%
O	31.7	O	40.81	O	40.11
W	29.46	In	20	In	20.7
In	21.02	Si	7.86	Si	6.92
Si	7.32	C	7.44	C	8.36
C	6.59	Cl	4.93	Cl	6.01
Sn	3.57	Zn	2.86	Zn	2.53
S	0.34	Sn	3.77	Sn	2.97
Total	100	S	3.71	S	2.99
		Al	5.43	Al	6.00
		W	3.18	W	3.41
		Total	100	Total	100

Table S2 compares the elemental composition of electrochromic devices during their life cycle. The pristine sample shows elements W from the tungsten oxide and In, Sn from the ITO layer. Si arises from the glass substrate. Trace amounts of C are atmospheric impurities. Chlorine and Sulphur species are part of the electrolyte. The first cycle shows an atomic relation of 4.6:1 Al to Zn, while the post mortem electrode shows a relation of 5.4:1 of the two species.

Table S3 shows the results of EDX analysis of the hydrogel used in the experiment in table S2. The relative amount of Al to Zn increases from 1.16:1 to 1.21:1.

Table S3: EDX analysis of hydrogel samples. Weight percentage of elements in a pristine hydrogel and the one used in cycling an electrochromic device. Both samples were initially soaked in 1M ZnAl electrolyte.

Pristine		Post Mortem	
Element	Wt%	El	Wt%
C	30.87	C	29.39
O	21.98	O	20.82
Na	0.77	Na	0.23
Al	2.93	Al	4.76
S	26.84	S	25.09
Cl	10.78	Cl	10.52
Zn	5.83	Zn	9.09
Total	100.00	Total	100.00

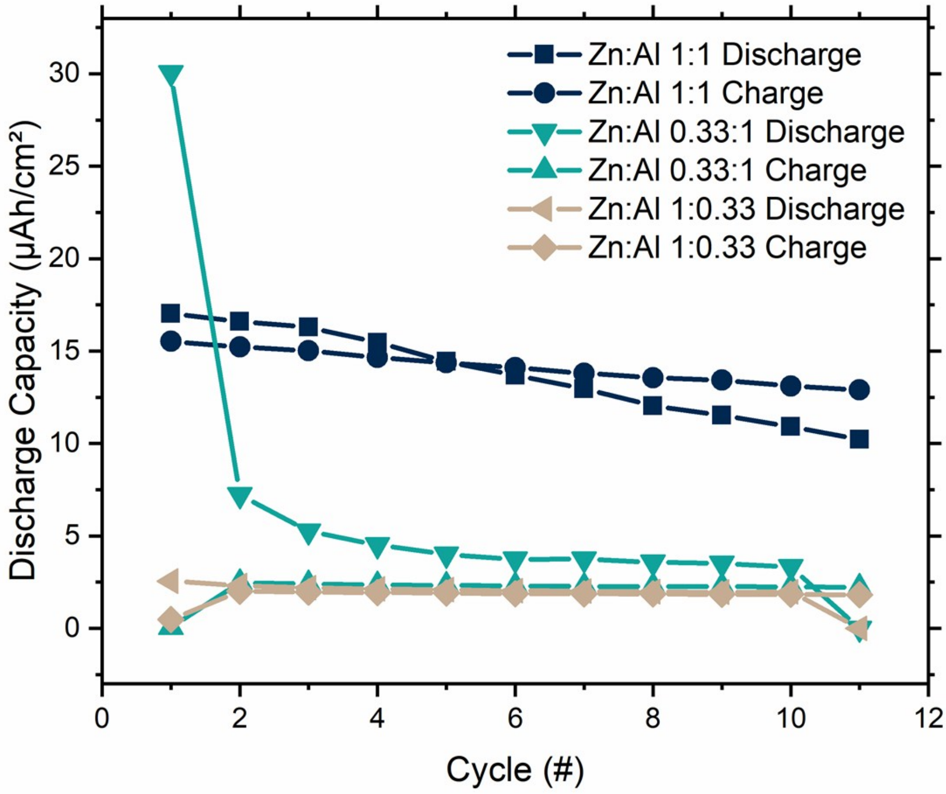

Fig. S12: Comparison of charge-discharge capacity for dual-ion electrochromic devices in different concentrations of electrolyte. The applied current density is 200 µA/cm². Electrolyte concentration were varied between 1 M Zn: 1M Al to 0.33 M Zn: 1 M Al and 0.33 M Al to 1 M Zn.

Fig. S12 depicts the dependence of charge capacity on the electrolyte concentration. The best charge capacity is achieved for a balanced electrolyte, while high relative concentrations of Al^{3+} lead to high irreversible effects, due to stripping of the Zn electrode. For high amounts of Zn ions, the overall capacity is low due to poor intercalation into WO_3.

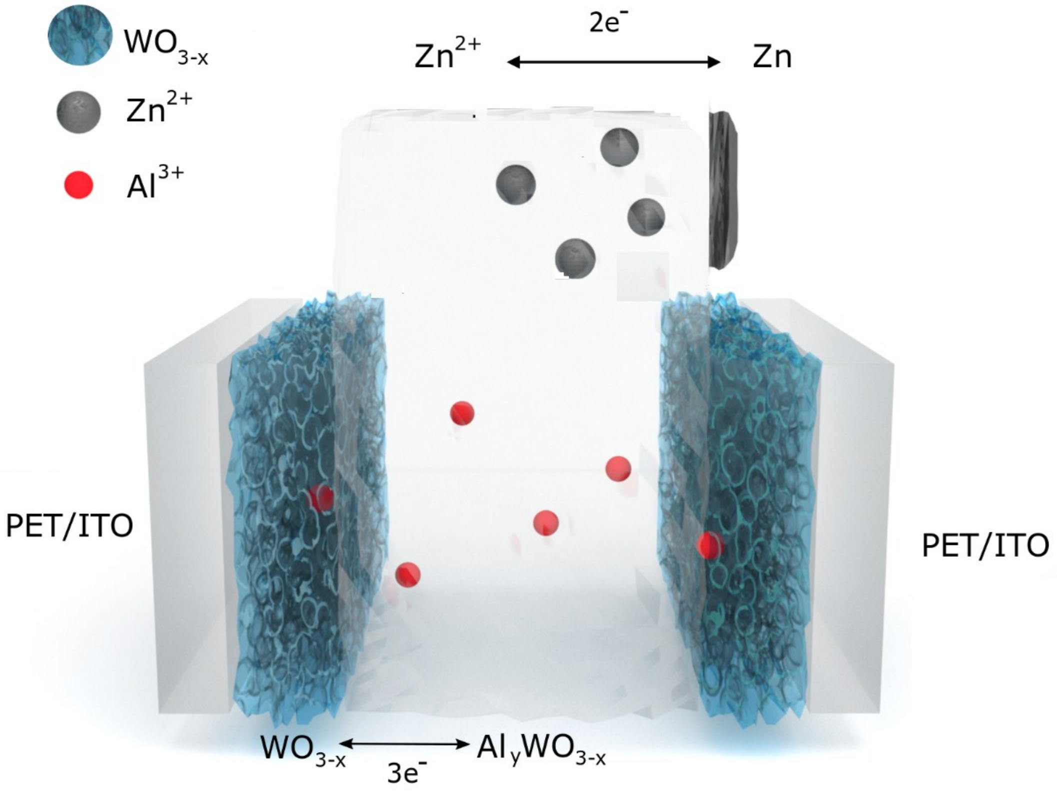

Fig. S13: Modified schematic for wearable prototype device. The zinc strip is attached to the side of the device to prevent skin contact. The device presented in Fig. 1, was modified for the case of a wearable device to prevent skin contact with zinc electrode and induce possible corrosion reactions.

Fig. S13 shows the modified schematic of the hydrogel-based device to prevent skin contact of the Zn electrode.

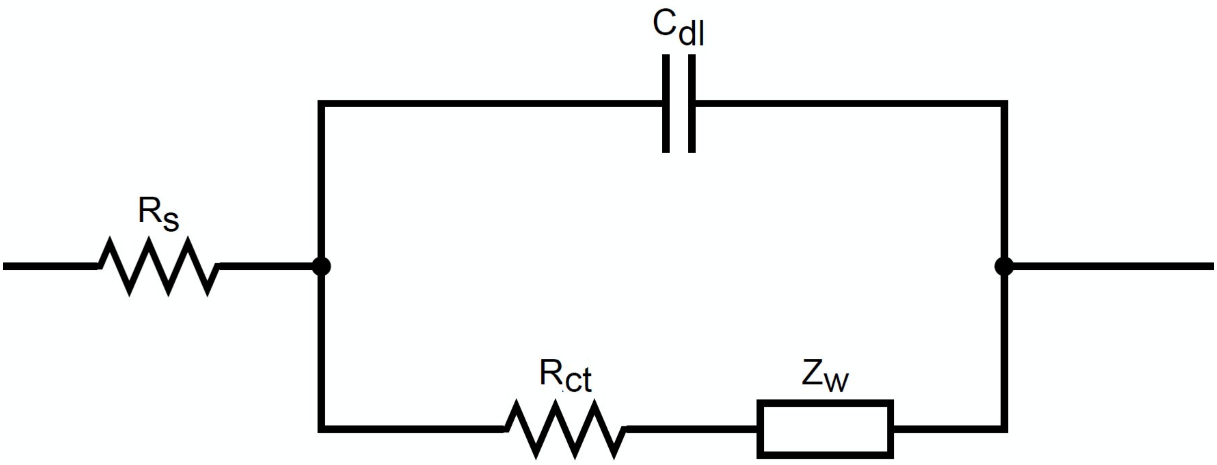

Figure S14: Randles cell equivalent circuit for WO_3-zinc electrochromic device.

Fig. S14 illustrates the equivalent circuit used to determine the ionic conductivity of devices. Here, R_s is the series resistance of the cables and the electrolyte, R_{ct} is the Faradaic charge-transfer resistance at the electrolyte/electrode interface, C_{dl} is the double-layer capacitance at the interface, and Z_w is the Warburg impedance which models ion diffusion into electrodes.

1. Mahajan, P. S., Tanpure, S. D., More, N. A., Gajbhiye, J. M. & Mhaske, S. B. Ammonium persulfate activated DMSO as a one-carbon synthon for the synthesis of methylenebisamides and other applications. *RSC Adv.* **5,** 101641–101646 (2015).

2. Limaye, M. V *et al.* Correlation between electrochromism and electronic structures of tungsten oxide films. *RSC Adv.* **4,** 5036 (2014).

3. Li, J. *et al.* A single-walled carbon nanotubes/poly(3,4-ethylenedioxythiophene)-poly(styrenesulfonate)/copper hexacyanoferrate hybrid film for high-volumetric performance flexible supercapacitors. *J. Power Sources* **386,** 96–105 (2018).
